# Optical coherence tomography-guided versus angiography-guided percutaneous coronary intervention: A meta-analysis of randomized controlled trials

**DOI:** 10.1016/j.ijcha.2024.101405

**Published:** 2024-04-05

**Authors:** Mushood Ahmed, Hira Javaid, Muhammad Talha Maniya, Aimen Shafiq, Haania Shahbaz, Priyansha Singh, Hritvik Jain, Jawad Basit, Mohammad Hamza, Abdulqadir J. Nashwan, Shafaqat Ali, Karthik Vadamalai

**Affiliations:** a Department of Medicine, Rawalpindi Medical University, Rawalpindi, Pakistan; b Department of Medicine, Allama Iqbal Medical College, Lahore, Pakistan; c Department of Medicine, Ziauddin University, Karachi, Pakistan; d Department of Medicine, Dow University of Health Sciences, Karachi, Pakistan; e Smt. Nathiba Hargovandas Lakhmichand Municipal Medical College, Ahmedabad, Gujarat, India; f Department of Internal Medicine, All India Institute of Medical Sciences (AIIMS), Jodhpur, India; g Cardiovascular Analytics Group, Canterbury, UK; h Guthrie Medical Group, Cortland, NY, USA; i Department of Nursing, Hamad Medical Corporation, Doha, Qatar; j Department of Medicine, Louisiana State University, Shreveport, LA USA; k University of Houston/HCA Kingwood Hospital, TX, USA

**Keywords:** Angiography, Optical coherence tomography, Percutaneous coronary intervention

## Abstract

**Background:**

Optical Coherence Tomography (OCT), a high-resolution imaging modality, guides stent implantation during percutaneous coronary intervention (PCI). However, OCT-guided PCI safety and efficacy data is limited.

**Methods:**

MEDLINE, Embase, and Cochrane Central were searched for randomized controlled trials (RCTs) comparing OCT-guided PCI to Angiography-guided PCI from inception to August 2023. A random-effects model was used to pool risk ratios (RRs), mean differences (MDs), and 95% confidence intervals (CIs) for clinical endpoints.

**Results:**

Our analysis included 5,139 patients from 11 studies. OCT-guided PCI resulted in a higher minimum stent area (MD = 0.35 [95 % CI, 0.21–0.49]; *p* < 0.00001), significantly reduced risk of cardiovascular mortality (RR = 0.56 [95 % CI, = 0.32–0.99]; *p* = 0.04), stent thrombosis (RR = 0.56 [95 % CI, 0.32–0.96]; *p* = 0.04), stent malapposition RR = 0.79 [95 % CI, 0.71–0.88]; *p* = < 0.0001) and major edge dissection (RR = 0.47 [95 % CI, 0.34–0.65]; *p* = <0.00001). However, no statistically significant difference was observed for all-cause mortality (RR = 0.71; *p* = 0.06), major adverse cardiovascular events (MACE) [RR = 0.80; *p* = 0.10], myocardial infarction (MI) [RR = 0.84; *p* = 0.16], target lesion revascularization (TLR) [RR = 0.94; *p* = 0.68], and target vessel revascularization (TVR) [RR = 0.91; *p* = 0.52].

**Conclusion:**

OCT-guided PCI led to an increased MSA and decreased cardiovascular mortality, stent thrombosis, stent malapposition, and major edge dissection. The incidence of all-cause mortality, MACE, MI, TLR, and TVR remained comparable across the two groups.

## 1 Introduction

Percutaneous coronary intervention (PCI) is regarded as the mainstay in treating coronary artery disease [1]. Although the limitations of angiography-guided PCI have been reported since the early 1990s, it remains the most used technique in clinical practice [2]. Despite its cost-effective nature, coronary angiography has undesired drawbacks, such as the inability to assess plaque severity and its characteristics accurately [3]. Intravascular imaging with optical coherence tomography (OCT) is an emerging approach that can be used to guide PCI.

OCT is a light-based cross-sectional imaging modality that offers higher resolution and faster imaging acquisition [4]. The higher resolution in OCT allows for more accurate differentiation of plaque characteristics and the detection of post-PCI complications such as stent malapposition and major edge dissections [5], [6]. Randomized controlled trials (RCTs) have been conducted in the past to evaluate the safety and efficacy of OCT-guided PCI as compared to conventional angiography-guided PCI [7], [8], [9]. However, most trials had small sample sizes and were not statistically powered to guide clinical practice.

Recently two large multicentric trials: OCTOBER (Optical Coherence Tomography Optimized Bifurcation Event Reduction) [10] and ILUMIEN IV (Optical Coherence Tomography Guided Coronary Stent Implantation Compared with Angiography) [11] published their findings. The trials showed conflicting results regarding the clinical outcomes following OCT-guided vs. angiography-guided PCI. The OCTOBER trial showed a reduced risk of major adverse cardiovascular events (MACE) at a 2-year follow-up with OCT-guided PCI. But in the ILUMIEN 4 trial, although the minimum stent area (MSA) was increased with OCT guidance, no significant difference was observed for reduction in all-cause mortality and target vessel failure.

Considering these conflicting findings, we conducted a comprehensive *meta*-analysis of all RCTs published to date to evaluate the effect of OCT-guided PCI on clinical outcomes. Moreover, the procedural safety of both PCI techniques was also analyzed.

## 2 Methods

The systematic review and *meta*-analysis followed the guidelines established by the Preferred Reporting Items for Systematic Review and Meta-Analysis (PRISMA) [12].

**Data sources and search strategy**

Two independent reviewers (P.S. and H.J.) systematically searched the literature from the PubMed/MEDLINE, Cochrane, and EMBASE databases. The search included RCTs published from the inception of these databases till September 2023. The objective was to identify all eligible studies that evaluated the efficacy and safety of OCT-guided PCI compared to angiography-guided PCI. Additional articles were identified by manually screening the reference list of retrieved trials, previous *meta*-analyses, and review articles to include all potentially pertinent publications. Redundant studies were excluded from the analysis employing Endnote X7 (Clarivate Analysis, PA). Moreover, in instances where multiple studies reported data from a common source or databank, only the study with the most participants was included in this *meta*-analysis. There were no restrictions instituted on the use of language. Detailed search strategies used for one of the databases are provided in Supplementary Table 1.

**Study selection and outcomes assessed**

The studies were considered eligible for inclusion in our systematic review and *meta*-analysis if they satisfied the following criteria: (a) were published RCTs or follow-up reports of RCTs; (b) included adult male or female (≥18 years of age) participants; (c) compared OCT-guided PCI to angiography-guided PCI; and (d) reported at least one of the selected pre-specified efficacy and safety outcomes. Excluded from our analysis were studies that needed more data, studies whose data did not align with our objectives, letters and editorials, case reports, case series, and reviews.

The primary outcomes included: (1) MSA, (2) All-cause and cardiovascular mortality. The secondary outcomes included MACE, stent thrombosis, myocardial infarction (MI), target lesion revascularization (TLR), target vessel revascularization (TVR), stent malapposition, and major edge dissection.

**Data extraction and quality assessment**

Two independent reviewers (H.J. and H.S.) evaluated the articles, and only those that satisfied the predetermined criteria were selected. Disagreements related to data were resolved by discussion, referring to the original article or opinion of the third reviewer (M.A.). The initial selection of papers was based on the title and abstract, followed by an in-depth review of the full text to verify their relevance. The extracted data encompassed various study characteristics, including the first author's surname, study design, publication year, sample size, duration of follow-up, outcomes, and demographic details of the participants, such as age, gender, and comorbidities like diabetes, hypertension, and previous myocardial infarction. The quality assessment of the studies included in this *meta*-analysis was conducted using the Cochrane risk of bias tool [13]. The Cochrane risk of bias tool encompasses the following aspects: selection bias, performance bias, attrition bias, detection bias, reporting bias, and other potential biases. Two reviewers (A.S. and M.T.M.) conducted the bias assessment independently. When discrepancies arose, a consensus was achieved through discussion between the two authors.

**Statistical analysis**

The statistical analysis was conducted using RevMan, version 5.4 (Nordic Cochrane Center, Copenhagen, Denmark), and Stata, version 17.0 (College Station, TX: StataCorp LLC). The research findings were presented as risk ratios (RRs) and mean difference (MD) for the dichotomous and continuous outcomes respectively, along with their corresponding 95 % confidence intervals (CIs), and the random effects model was used to pool results [14]. To visually evaluate the outcomes of pooling, forest plots were generated. Publication bias was evaluated by performing a visual inspection of funnel plots. The Higgins I^2^ values were utilized to assess heterogeneity due to variations in study methodologies and populations [15]. A *meta*-regression analysis was performed for the primary outcomes to determine the effect of follow-up duration and publication year on the pooled estimates calculated using a random effects DerSimonian-Laird model in our analysis. Meta-regression bubble plots were generated to visually evaluate the results of the *meta*-regression analysis. The detailed results were tabulated. A p-value of less than 0.05 was considered statistically significant in all cases.

## 3 Results

**Results of Literature Search**

**Fig. 1:**
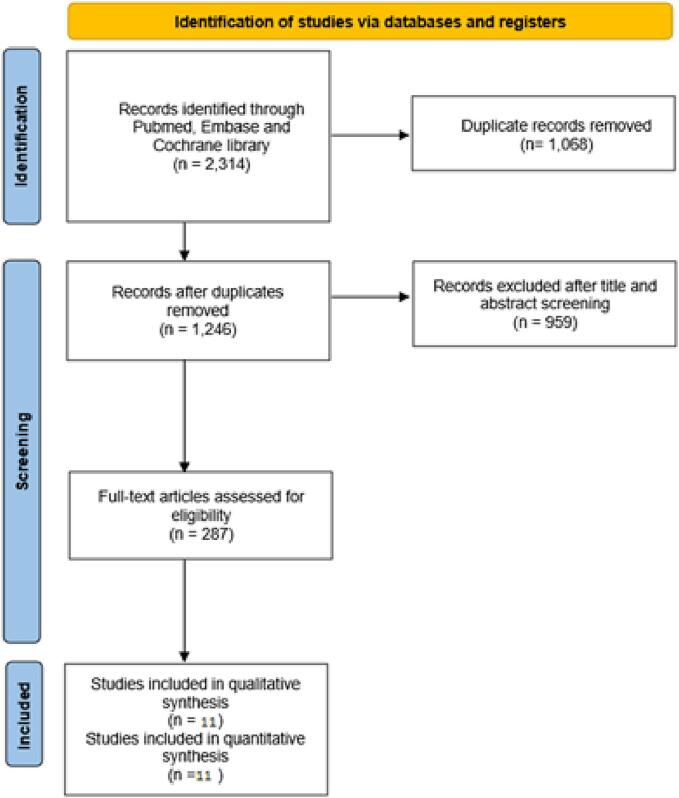
PRISMA flowchart summarizing the study selection process.

The systematic literature search yielded a total of 2314 records. A full-text review of 287 studies identified 11 eligible studies [7], [8], [9], [10], [11], [16], [17], [18], [19], [20], [21] in line with our inclusion and exclusion criteria, which were ultimately included in the analysis. The PRISMA flowchart presented in Fig. 1 outlines the systematic literature search and study selection process.

**Study characteristics and quality assessment**

A total of 5,139 participants were included, of which there were 2,575 in the OCT group and 2,564 in the angiography group. All studies had a higher proportion of male patients than female patients. The mean age of patients in the included studies ranged from 57 to 70 years old. There was variability in the clinical presentation of the patients undergoing PCI. Studies enrolled patients with coronary artery disease and/or acute coronary syndromes. The baseline characteristics and study designs of the included studies are presented in Table 1.

**Table 1:** Baseline characteristics of the included studies and participants.

**Author/Study name**	**Study design**	**Year**	**OCT guided PCI (n)**	**AG guided PCI (n)**	**Males (%)**	**Patient Baselines**				**Indication for PCI**	**Follow up**
						**Mean age (SD/IQR)**	**Diabetes (%)**	**Hypertension (%)**	**Previous MI (%)**		
ILUMIEN IV	RCT	2023	1233	1254	OCT 78.5 %	OCT 65.5 ± 10.5	OCT 42.4 %	OCT71.4 %	OCT 20.4 %	CAD	2 years
					AG 76.2 %	AG 65.7 ± 10.3	AG 41.5 %	AG 74.0 %	AG 24.2 %		
OCTOBER	RCT	2023	600	601	OCT 78.8 %	OCT 66.4 ± 10.5	OCT 17.2 %	OCT 70.3 %	OCT 28.3 %	Stable angina and ACS	2 years
					AG 79 %	AG 66.2 ± 9.9	AG 16.1 %	AG 74.5 %	AG 30 %		
Kim et al.*	RCT	2015	58	59	OCT 78 % AG 72.5 %	OCT 58.8 ± 10.8 AG 61.6 ± 9.7	OCT 32 % AG 31.4 %	OCT 54 % AG 49 %	OCT 6 % AG 2 %	Stable angina and ACS	12 months
iSIGHT	RCT	2021	51	49	OCT 60.8 %	OCT 59.92 ± 8.92	OCT 33.3 %	OCT 90.2 %	OCT29.4 %	Stable angina and ACS	12 months
					AG 77.5 %	AG 58.59 ± 10.20	AG 44.9 %	AG 79.6 %	AG 34.7 %		
ROBUST	RCT	2018	105	96	OCT 83 %	OCT 57(46–70)	OCT17%	OCT 50 %	OCT 1 %	STEMI	9 months
					AG 87 %	AG 59(47–72)	AG 26 %	AG 52 %	AG 6 %		
DOCTORS	RCT	2016	120	120	OCT 79.2 %	OCT 60.8 ± 11.5	OCT 21.7 %	OCT 55.8 %	NR	NSTE-ACS	6 months
					AG 75.8 %	AG 60.2 ± 11.3	AG 15.8 %	AG 41.7 %			
ILUMIEN III (short-term follow-up)	RCT	2016	158	146	OCT 69 %	OCT 66 (59–72)	OCT33%	OCT 78 %	OCT 22 %	Coronary heart disease	30 days
					AG 73 %	AG 67 (56–65)	AG 42 %	AG 75 %	AG 22 %		
OCTACS	RCT	2015	50	50	OCT 72 %	OCT 61.8 ± 9.4	OCT16%	OCT 56 %	OCT 4 %	NSTEMI-ACS	6 months
					AG 68 %	AG 62.6 ± 11.0	AG 10 %	AG 56 %	AG 0 %		
OPTICO BVS	RCT	2020	19	19	OCT 79 %	OCT 63.3 ± 12.7	OCT 21 %	OCT 63 %	NR	Stable angina and ACS	6 and 12 months
					AG 79 %	AG 62.9 ± 9.1	AG 21 %	AG 63 %	NR		
OPTICO-Integration II	RCT	2020	28	28	72.6 %	70 (62–78)	23.8 %	83.3 %	NR	CAD	NR
ILUMIEN III (long-term follow-up)	RCT	2021	153	142	OCT 69 %	OCT 66 (59–72)	OCT 33 %	OCT 78 %	NR	Coronary heart disease	12 months
					AG 73 %	AG 67 (56–75)	AG 28 %	AG 75 %			

AG: Angiography, ACS: Acute coronary syndrome, CAD; Coronary artery disease, IQR: Interquartile range, MI: Myocardial Infraction, NSTEMI; Non-ST elevated myocardial infraction, NSTE-ACS; Non-ST elevated acute coronary syndromes n: number, NR: Not reported, OCT: Optical coherence tomography, PCI: Percutaneous coronary intervention, RCT: Randomized controlled trial, STEMI; ST-elevated myocardial infraction SD: Standard deviation

* The trial has provided detailed patients’ characteristics for the participants who underwent OCT follow-up assessment at 6 months for the percentage of uncovered struts.

All studies were of reasonably high methodological quality. The details of the risk of bias assessment for the included RCTs are provided in Supplementary Figs. 1-2. A summary of the results of the *meta*-regression analysis can be found in Supplementary Table 2. The visual inspection of funnel plots did not show a considerable risk of publication bias (Supplementary Figs. 3-5**)**.

## 4 Results of meta-analysis

*MSA*

**Fig. 2:**
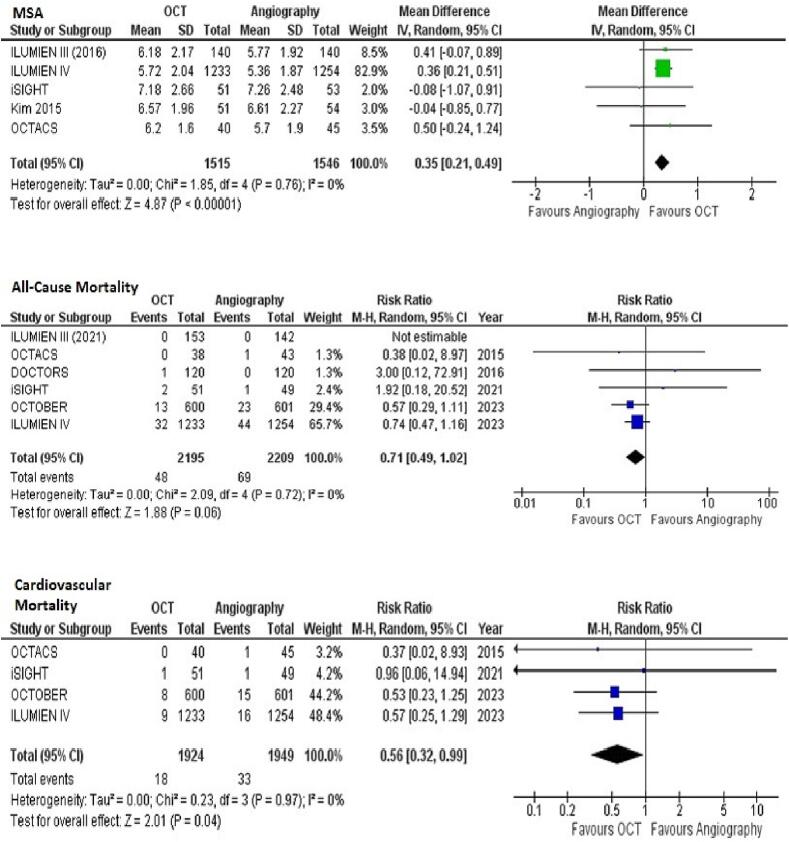
Forest plots for (A) minimum stent area (B) All-cause mortality (C) Cardiovascular mortality with OCT-guided versus angiography-guided PCI.

Data on MSA was reported in 5 studies. The pooled analysis demonstrated a significantly increased MSA with OCT-guided PCI compared to angiography-guided PCI (MD = 0.35 [95 % CI, 0.21–0.49]; *p* < 0.00001; Fig. 2A). Heterogeneity between study results was non-significant (I^2^ = 0 %). Meta-regression analysis was not significant for publication year (*p* = 0.7002; Supplementary Figure 6) or follow-up (*p* = 0.7211; Supplementary Figure 7) duration.

***All-Cause Mortality***

Data on all-cause mortality was reported in 6 of 11 included studies. The pooled estimates favored OCT-guided PCI but failed to demonstrate a statistically significant reduced risk of all-cause mortality with OCT-guidance as compared to angiography-guided PCI (RR = 0.71 [95 % CI, 0.49–1.02]; *p* = 0.06; Fig. 2B). No heterogeneity was observed (I^2^ = 0 %). Meta-regression analysis was not significant for publication year (*p* = 0.9492; Supplementary Figure 8) or follow-up (*p* = 0.4446; Supplementary Figure 9) duration.

***Cardiovascular Mortality***

Data on cardiovascular mortality was reported in 4 of 11 included studies. The pooled analysis demonstrated a significantly reduced risk of cardiovascular mortality with OCT-guided PCI compared to angiography-guided PCI (RR = 0.56 [95 % CI, = 0.32–0.99]; *p* = 0.04 Fig. 2C). The heterogeneity was not significant (I^2^ = 0 %). Meta-regression analysis was not significant for publication year (*p* = 0.8920; Supplementary Figure 10) or follow-up (*p* = 0.9683; Supplementary Figure 11) duration.

***MACE***

**Fig. 3:**
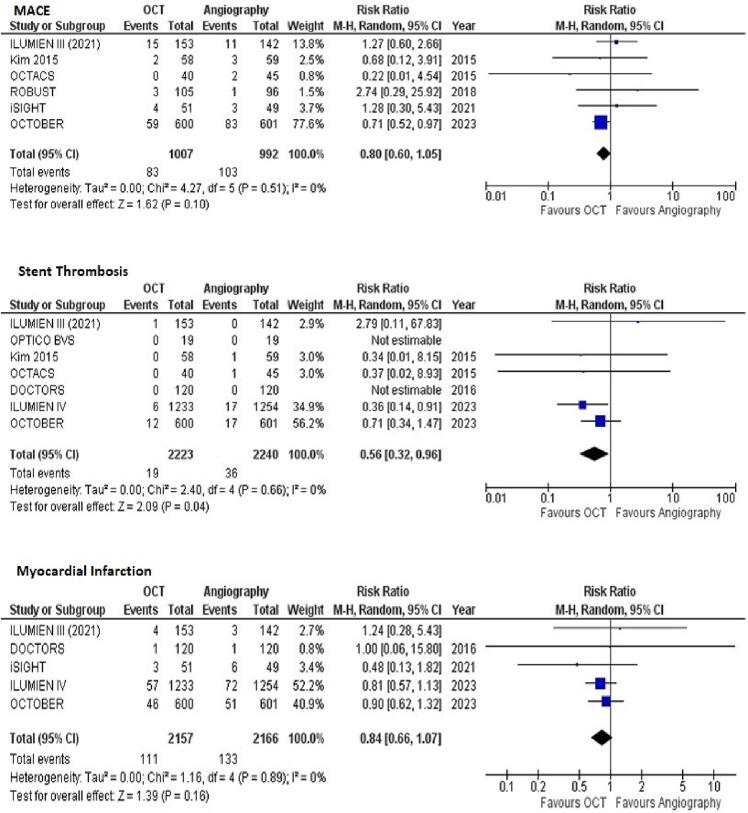
Forest plots for (A) MACE (B) Stent thrombosis (C) Myocardial Infarction with OCT-guided versus angiography-guided PCI.

Data on MACE was reported in 6 of 11 included studies. The pooled analysis demonstrated no statistically significant reduced risk of MACE with OCT-guided PCI compared to Angiography-guided PCI. (RR = 0.80 [95 % CI, 0.60–1.05]; *p* = 0.10; Fig. 3A). There was no heterogeneity between study results (I^2^ = 0 %).

***Stent Thrombosis***

Data on stent thrombosis was reported in 7 of 11 included studies. The pooled analysis demonstrated a significantly reduced risk of stent thrombosis with OCT-guided PCI compared to angiography-guided PCI (RR = 0.56 [95 % CI, 0.32–0.96]; *p* = 0.04; Fig. 3B). There was no heterogeneity between study results (I^2^ = 0 %).

***Myocardial Infarction***

Data on myocardial infarction (due to any cause) was reported in 5 of 11 included studies. The pooled analysis demonstrated no significantly reduced risk of myocardial infarction with OCT-guided PCI compared to angiography-guided PCI (RR = 0.84 [95 % CI, 0.66–1.07]; *p* = 0.16; Fig. 3C). There was no heterogeneity between study results (I^2^ = 0 %). The point estimates for procedural MI were comparable across two groups (RR = 0.80 [95 % CI, 0.56–1.15]; *p* = 0.23; Supplementary Figure 12).

*TLR*

**Fig. 4:**
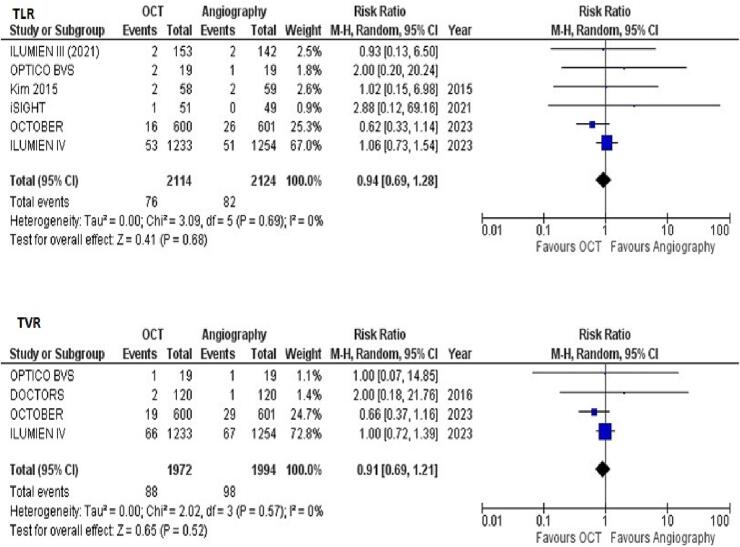
Forest plots for (A) TLR (B) TVR with OCT-guided versus angiography-guided PCI.

Data on TLR was reported in 6 of 11 included studies. The pooled analysis demonstrated no significant difference in risk of TLR between OCT-guided PCI versus angiography-guided PCI (RR = 0.94 [95 % CI 0.69–1.28]; *p* = 0.68; Fig. 4A). There was no heterogeneity between study results (I^2^ = 0 %).

*TVR*

Data on TVR was reported in 4 of 11 included studies. The pooled analysis demonstrated no significant difference in risk of TVR between OCT-guided PCI versus angiography-guided PCI (RR = 0.91 [95 % CI, 0.69–1.21]; *p* = 0.52; Fig. 4B). There was no heterogeneity between study results (I^2^ = 0 %).

**Stent malapposition and edge dissection**

**Fig. 5:**
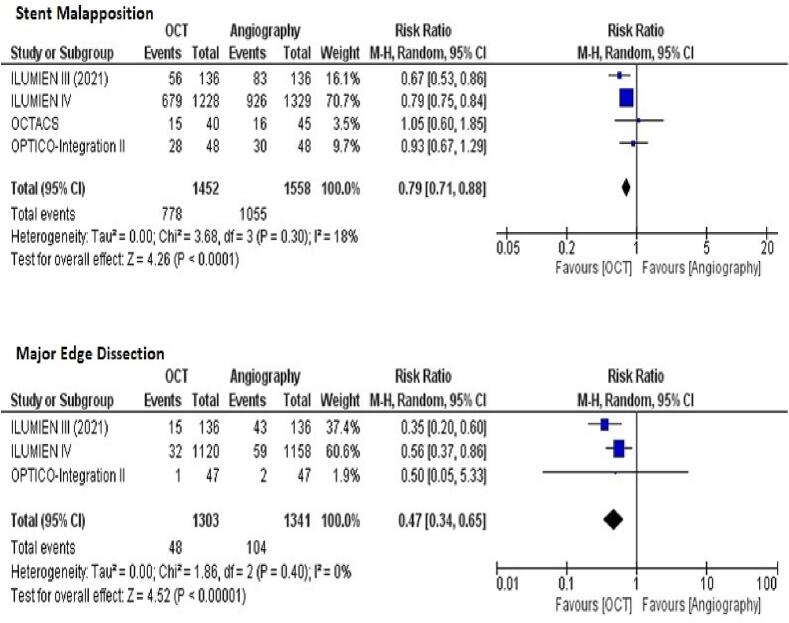
Forest plots for (A) Stent malapposition (B) Major edge dissection with OCT-guided versus angiography-guided PCI.

The pooled analysis demonstrated a significantly reduced risk of stent malapposition with OCT-guided PCI as compared to angiography-guided PCI (RR = 0.79 [95 % CI, 0.71–0.88]; *p* = < 0.0001; Fig. 5A). The heterogeneity between study results was not significant (I^2^ = 18 %).

Data regarding edge dissection was reported by 4 of the 11 included studies. OCT guidance was associated with a statistically significant reduced risk of major edge dissection (RR = 0.47 [95 % CI, 0.34–0.65]; *p* = <0.00001; Fig. 5B). No significant heterogeneity was observed (I^2^ = 0 %).

## 5 Discussion

In this comprehensive *meta*-analysis, we compare OCT-guided PCI to angiography-guided PCI, highlighting many significant findings. Firstly, OCT-guided PCI was reported to demonstrate significantly increased MSA compared to angiography-guided PCI. Moreover, OCT-guided PCI significantly reduced the risk of cardiovascular mortality, stent thrombosis, stent malapposition, and major edge dissection. However, no significant difference was observed in all-cause mortality, MACE, myocardial infarction, TLR, and TVR with OCT-guided PCI relative to angiography-only PCI. We observed no significant effect of follow-up duration and publication year on the pooled risk ratios for the primary outcomes.

The definitive indicator of stent success is its conclusive MSA [22]. In the ILUMIEN I study [23], OCT application resulted in the selection of smaller diameter stents in around one-third of patients compared to those where angiography alone was employed. The lack of visualization of the actual vessel size (defined by the external elastic lamina [EEL] at the lesion site on OCT) was presumably the cause of this observation since the light only penetrated a limited depth through lipid-rich plaque [24], [25]. As a result, stent size assessment and lumen-guided diameter measurement emerged as the standard procedure using OCT guidance. Although several *meta*-analyses [26], [27], [28], [29], [30], [31], [32], [33], [34] have been done in the past only Siddiqi et al. [35] discovered that OCT-guided PCI is strongly correlated with a substantial tendency towards a bigger MSA. However, the results were not proven to be statistically significant. In contrast, our *meta*-analysis concludes a statistically significant increase in MSA in OCT-guided PCI compared to angiography alone. This is in contrast to the previous findings. These results were achieved due to the inclusion of a recently published trial [11], having a relatively more extended follow-up period, and demonstrating a higher MSA with OCT-guided PCI. Hence, greater MSAs obtained following OCT guidance suggest the enhanced efficiency of its modality for directing PCI in clinical practice.

The previously conducted *meta*-analyses have reported conflicting findings regarding the impact of intravascular imaging on mortality and ST [32], [34], [35], [26], [27], [28], [29], [30]. In our pooled analysis, we have exclusively focused on OCT vs. angiography-guided PCI. This is in contrast to a number of recent *meta*-analyses [26], [27], [28] which synthesized evidence from several RCTs in which intravascular ultrasound (IVUS) guidance was used for stent implantation. Narrowing our scope to OCT ensures homogeneity in the intervention method and a focused target analysis. This meticulous approach allowed robust and comprehensive assessment of clinical outcomes following OCT-guided PCI which can provide valuable evidence for the applicability of OCT-guided PCI in clinical practice as compared to routinely used angiography-guided PCI.

Our *meta*-analysis evaluated all RCTs published to date and demonstrated that the use of OCT-guided PCI reduced cardiovascular mortality and pooled estimates favored OCT-imaging for reducing all-cause mortality. This can be attributed to a decreased risk of stent thrombosis with OCT imaging. Stent thrombosis is the most severe PCI-specific stent complication [36]. In the ILUMIEN 4 trial [11], 95.7 % of patients with stent thrombosis suffered an MI and died. Hence, the reduced risk of stent thrombosis following an OCT-guided PCI is particularly beneficial and aids in deciding between OCT-guided versus angiography-alone techniques of PCI.

OCT imaging appeared to improve procedural outcomes that included reduced risk of stent malapposition and major edge dissection. These findings demonstrate the procedural superiority of OCT imaging. These findings are unique as they were not analyzed in earlier *meta*-analyses [32], [33], [35], [26], [27], [28], [29], [30]. However, the additional adverse effects of stents, including MI, TLR, and TVR are anticipated to decrease due to the larger MSA obtained through OCT compared to angiography alone. But the rates of MI, TVR, and TLR were comparable between the two groups, possibly due to the low occurrence rates of these events and, consequently, the reduced statistical ability to identify any minor differences. The results of our *meta*-analysis should be considered hypothesis-generating and evidence from new RCTs that are specifically designed to evaluate the impact of stent-related outcomes with OCT guidance is required for a better understanding of these comparable rates observed in our pooled analysis.

Although there have been earlier *meta*-analyses our *meta*-analysis offers the most recent and comprehensive overview since it includes all studies performed to date, including those that earlier *meta*-analyses overlooked and those that have been published more recently. The *meta*-analysis by Sreenivasan at al. [27] included 6 RCTs that compared OCT with angiography-guided PCI (10 of the included RCTs compared IVUS guided PCI with angiography guided-PCI), Khan et al. [28] included 8 RCTs (12 of the included studies compared IVUS vs angiography-guided PCI), Meyer et al. [33] pooled 3 RCTs comparing OCT with angiography-guided PCI in ACS patients, Farah et al. [26] included 4 trials (10 RCTs reported data for IVUS vs. angiography guided PCI), Kuno et al. [29] included 32 RCTs in their analysis however, only 8 reported data for OCT and angiography-guided PCI, while Saylık et al. [32] and Park et al. [30] included 5 RCTs comparing OCT with angiography-guided PCI. The *meta*-analysis by Attar et al. [31] had methodological inaccuracies as well. The authors pooled the EROSION III [37] (A Multicenter RCT of OCT-Guided Reperfusion in STEMI With Early Infarct Artery Patency) trial in their *meta*-analysis. However, EROSION III focused on the rate of stent implantation in a subset of STEMI patients (presentation with < 70 % stenosis and TIMI III); where the OCT vs Angio guidance randomization was primarily involved in the decision of implanting a stent, not guiding the intervention. As a result, several patients in that study did not undergo stent implantation. Hence, pooling this RCT could have resulted in an overestimation of the effect sizes in the previous *meta*-analysis. In our study, we performed a rigorous and robust literature review to ensure that only those RCTs that evaluated PCI with OCT imaging and angiography alone were included. We also included iSIGHT [18] and OPTICO-integration II trials [19] not pooled by the previous *meta*-analysis. Moreover, we evaluated the procedural outcomes including stent malapposition and major edge dissection not analyzed in previous *meta*-analyses [38], [26], [27], [28], [29], [30], [31], [32], [33], [34], [35]. We also performed *meta*-regression for the primary outcomes to assess whether the study outcomes had a temporal trend or influenced by the duration of follow-up not analyzed in prior studies. Hence the current state of literature is assessed according to the highest degree of accuracy to guide clinicians regarding the efficacy of both imaging modalities.

The current *meta*-analysis has some limitations as well. Firstly, it was assumed that the baseline characteristics of those participating in the included studies would be comparable when performing this *meta*-analysis. However, the differences in patient characteristics and prior treatments may have fostered clinical heterogeneity. Although we analyzed a number of clinical outcomes, data for all assessed measures was only available from a limited number of the RCTs. Furthermore, OCT assessments of the included studies were conducted at random intervals and with relatively brief follow-up durations. Additionally, some of the studies were constrained by their small sample sizes.

## 6 Conclusion

The utilization of OCT in the guidance of PCI resulted in a notable increase in MSA and a corresponding decrease in cardiovascular mortality and stent thrombosis. It is crucial to acknowledge that despite the observed increase in MSA, the influence of OCT guidance did not demonstrate a statistically significant effect on all-cause mortality, MACE, MI, TLR, and TVR. Further evidence is still required to establish the clinical superiority of OCT-guided PCI over angiography-guided PCI.

**Guarantor of the article:** Mushood Ahmed, MBBS.

**Financial support:** No financial support received.

**Author contributions:**

Conceptualization, data curation and project administration were carried out by MA, JB and AJN.

Supervision was carried out by MA, AJN, MH and JB.

Formal analysis of data was carried out by MTM, AS, MA, KV and HJ.

Formal analysis, methodology, and software was carried out by MTM, HJ, and HS.

Writing the original draft was carried out by MA, HJ, AS, PS, MH and MTM.

Writing, reviewing, and editing was carried out by HJ, HS, PS, KV, SA and KJ.

Visualization and validation were carried out by MA, HJ, AJN, JB, AS and MTM.

## CRediT authorship contribution statement

**Mushood Ahmed:** Writing – original draft, Visualization, Validation, Supervision, Formal analysis, Data curation, Conceptualization. **Hira Javaid:** Writing – review & editing, Writing – original draft, Formal analysis. **Muhammad Talha Maniya:** Writing – original draft, Methodology, Formal analysis. **Aimen Shafiq:** Writing – original draft, Visualization, Validation, Formal analysis. **Haania Shahbaz:** Writing – review & editing, Software, Methodology, Formal analysis. **Priyansha Singh:** Writing – review & editing, Writing – original draft. **Hritvik Jain:** Writing – review & editing. **Jawad Basit:** Supervision, Project administration, Data curation, Conceptualization. **Mohammad Hamza:** Writing – original draft. **Abdulqadir J. Nashwan:** Visualization, Validation, Supervision, Data curation, Conceptualization. **Shafaqat Ali:** Writing – review & editing. **Karthik Vadamalai:** Writing – review & editing, Formal analysis.

## Declaration of competing interest

The authors declare that they have no known competing financial interests or personal relationships that could have appeared to influence the work reported in this paper.
