# Supplementary material for: Optical coherence tomography-guided versus angiography-guided percutaneous coronary intervention: A meta-analysis of randomized controlled trials
Source: Int J Cardiol Heart Vasc. 2024 Apr 5;52:101405. doi: 10.1016/j.ijcha.2024.101405 (PMC11156695; doi:10.1016/j.ijcha.2024.101405)

**Supplementary Appendix**

**Supplementary Table 1**: Search Methodology For MEDLINE/PubMed

**Supplementary Table 2:** Table summarizing results of regression analysis

**Supplementary Figure 1**: Summary of quality assessment.

**Supplementary Figure 2**: Risk of bias graph.

**Supplementary Figure 3**: Funnel plots for (A) minimum stent area (B) All-cause mortality (C) Cardiovascular mortality

**Supplementary Figure 4:** Funnel plots for (A) MACE (B) Stent thrombosis (C) Myocardial Infarction

**Supplementary Figure 5**: Funnel plots for (A) TLR (B) TVR (C) Stent malapposition (D) Major edge dissection

**Supplementary Figure 6:** Meta-regression plot assessing the effect of Publication Year on the risk ratios of Minimum Stent Area (MSA)

**Supplementary Figure 7:** Meta-regression plot assessing the effect of Follow-up on the risk ratios of Minimum Stent Area (MSA)

**Supplementary Figure 8:** Meta-regression plot assessing the effect of Publication Year on the risk ratios of All-Cause Mortality

**Supplementary Figure 9:** Meta-regression plot assessing the effect of Follow-up on the risk ratios of All-Cause Mortality

**Supplementary Figure 10:** Meta-regression plot assessing the effect of Publication Year on the risk ratios of Cardiovascular Mortality

**Supplementary Figure 11:** Meta-regression plot assessing the effect of Follow-up on the risk ratios of Cardiovascular Mortality

**Supplementary Table 1**: Search Methodology For MEDLINE/PubMed”

| **Term Number** | **Search Strategy** | **Number of Results** |
| --- | --- | --- |
|  | "Percutaneous Coronary Intervention"[Mesh] OR (Coronary Intervention, Percutaneous) OR (Coronary Interventions, Percutaneous) OR (Percutaneous Coronary Interventions) OR (Percutaneous Coronary Revascularization) OR (Coronary Revascularization, Percutaneous) OR (Coronary Revascularizations, Percutaneous) OR (Percutaneous Coronary Revascularizations) | 84,054 |
|  | “Tomography, Optical Coherence”[MeSH] OR (OCT Tomography) OR (Optical Coherence Tomography) OR (Tomography, Optical) | 153,415 |
|  | 1 AND 2  ("Percutaneous Coronary Intervention"[Mesh] OR (Coronary Intervention, Percutaneous) OR (Coronary Interventions, Percutaneous) OR (Percutaneous Coronary Interventions) OR (Percutaneous Coronary Revascularization) OR (Coronary Revascularization, Percutaneous) OR (Coronary Revascularizations, Percutaneous) OR (Percutaneous Coronary Revascularizations)) AND (“Tomography, Optical Coherence”[MeSH] OR (OCT Tomography) OR (Optical Coherence Tomography) OR (Tomography, Optical)) | 3027 |
|  | “Coronary Angiography”[MeSH] OR (Angiography, Coronary) OR (Angiographies, Coronary) | 105,700 |
|  | 3 AND 4  ("Percutaneous Coronary Intervention"[Mesh] OR (Coronary Intervention, Percutaneous) OR (Coronary Interventions, Percutaneous) OR (Percutaneous Coronary Interventions) OR (Percutaneous Coronary Revascularization) OR (Coronary Revascularization, Percutaneous) OR (Coronary Revascularizations, Percutaneous) OR (Percutaneous Coronary Revascularizations)) AND (("Tomography, Optical Coherence"[MeSH] OR (OCT Tomography) OR (Optical Coherence Tomography) OR (Tomography, Optical))) AND ("Coronary Angiography"[MeSH] OR (Angiography, Coronary) OR (Angiographies, Coronary)) | 1791 |

**Supplementary Table 2:** Table summarizing results of regression analysis

| Outcome | Co-efficient (95% Confidence Interval) | Intercept (95% Confidence Interval) | *p-value* |
| --- | --- | --- | --- |
| Minimum Stent Area | | | |
| Follow-Up (Months) | -0.0171972 (-0.1116316 to 0.0772372) | 0.3559228 (0.2120453 to 0.4998003) | 0.7211 |
| Publication Year | 0.0136118 (-0.0556832 to 0.0829068) | -27.17717 (-167.3089 to 112.9546) | 0.7002 |
| All-Cause Mortality | | | |
| Follow-Up (Months) | -0.0403117 (-0.1436648 to 0.0630414) | 0.5872994 (-1.832665 to 3.007264) | 0.4446 |
| Publication Year | 0.0121866 (-0.3626003 to 0.3869735) | -25.01613 (-783.1463 to 733.1141) | 0.9492 |
| Cardiovascular Mortality | | | |
| Follow-Up (Months) | -0.0029338 (-0.1477939 to 0.1419263) | -0.5129779 (-3.880799 to 2.854843) | 0.9683 |
| Publication Year | 0.0271121 (-0.3642357 to 0.41846) | -55.41888 (-846.9831 to 736.1453) | 0.8920 |

**Supplementary Figure 1**: Summary of quality assessment.


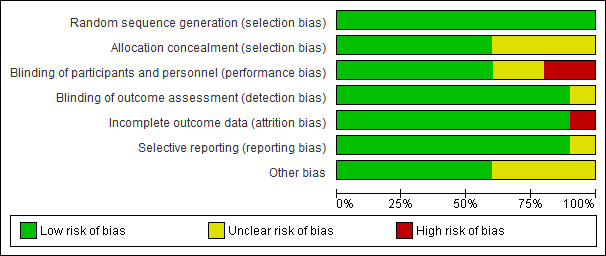


**Supplementary Figure 2**: Risk of bias graph.

**
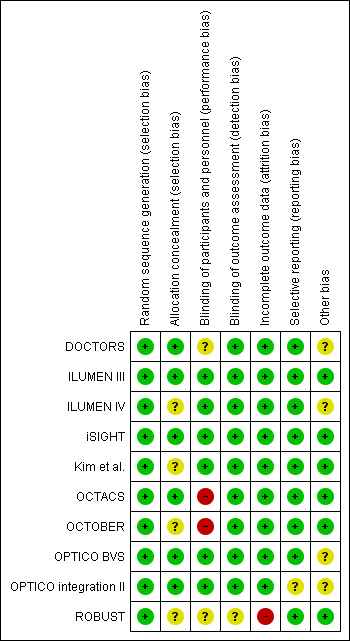
**

**Supplementary Figure 3**: Funnel plots for (A) minimum stent area (B) All-cause mortality (C) Cardiovascular mortality


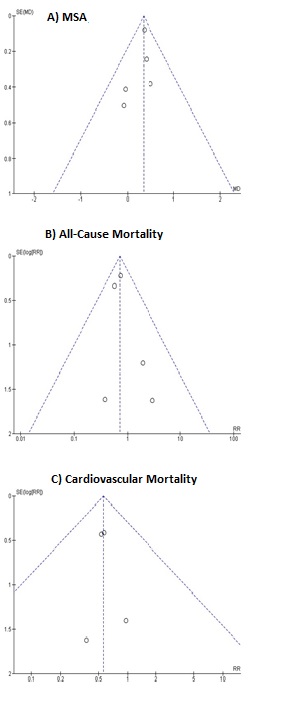


**Supplementary Figure 4**: Funnel plots for (A) MACE (B) Stent thrombosis (C) Myocardial Infarction


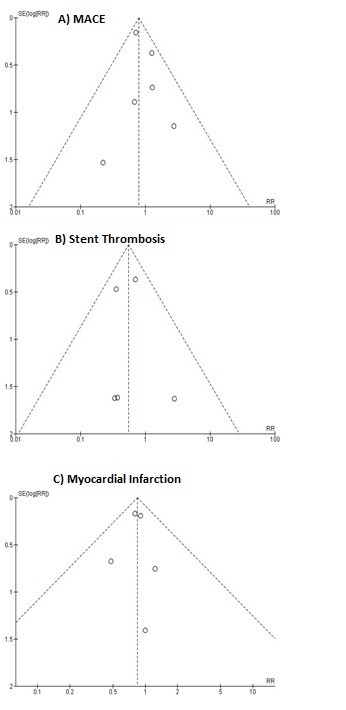


**Supplementary Figure 5**: Funnel plots for (A) TLR (B) TVR (C) Stent malapposition (D) Major edge dissection


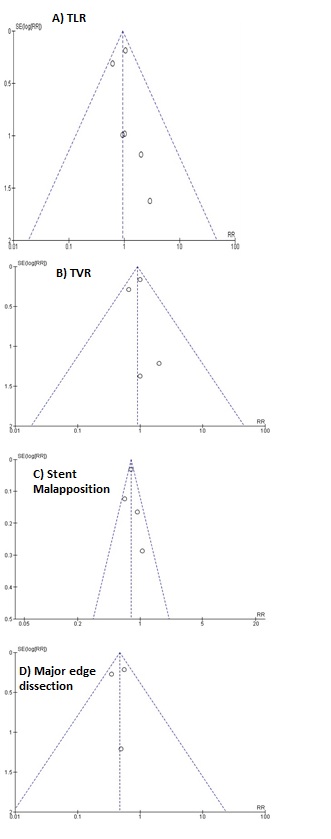


**Supplementary Figure 6:** Meta-regression plot assessing the effect of Publication Year on the risk ratios of Minimum Stent Area (MSA).


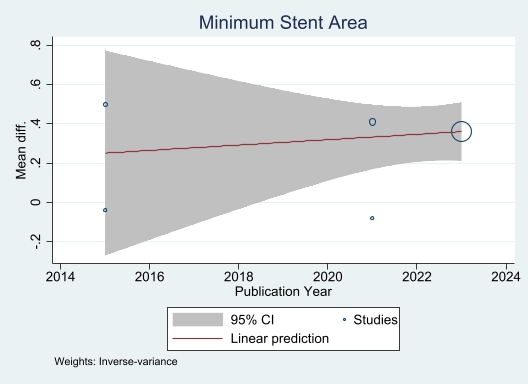


**Supplementary Figure 7:** Meta-regression plot assessing the effect of Follow-up on the risk ratios of Minimum Stent Area (MSA).


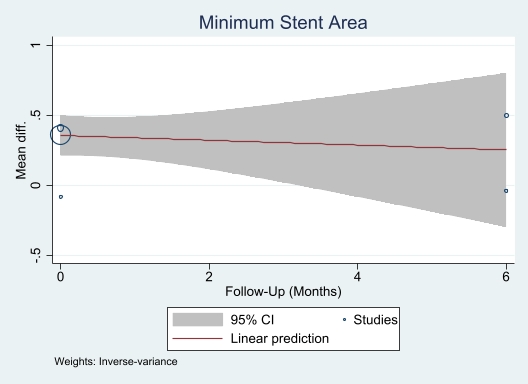


**Supplementary Figure 8:** Meta-regression plot assessing the effect of Publication Year on the risk ratios of All-Cause Mortality.


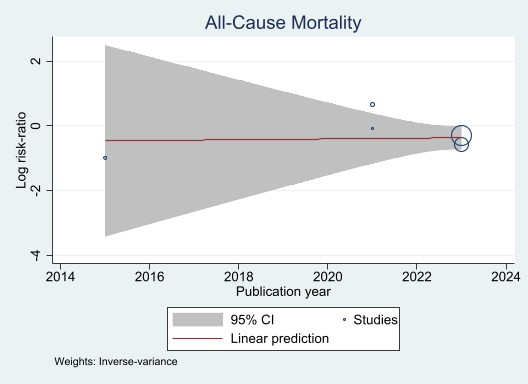


**Supplementary Figure 9:** Meta-regression plot assessing the effect of Follow-up on the risk ratios of All-Cause Mortality.


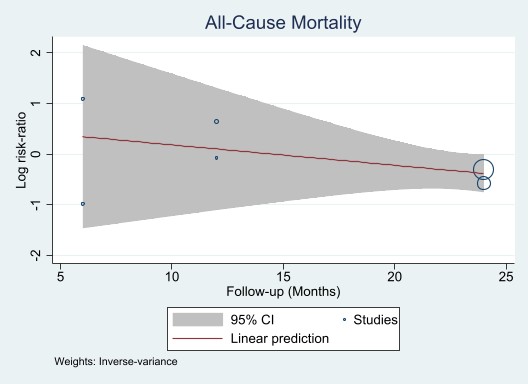


**Supplementary Figure 10:** Meta-regression plot assessing the effect of Publication Year on the risk ratios of Cardiovascular Mortality.


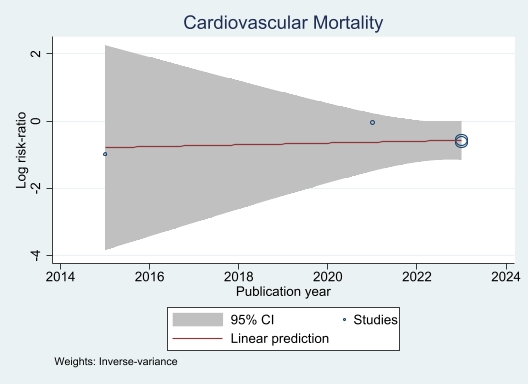


**Supplementary Figure 11:** Meta-regression plot assessing the effect of Follow-up on the risk ratios of Cardiovascular Mortality.


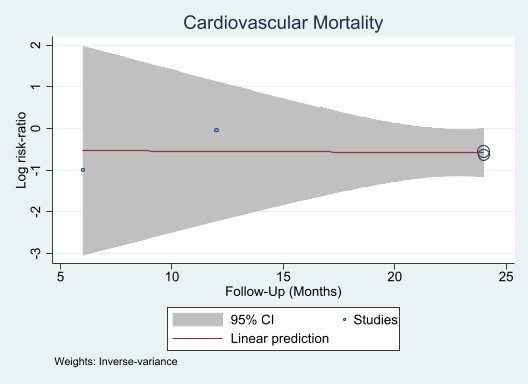

Supplement: Supplementary data 1 [file mmc1.docx]
